# Brain Death Secondary to Rocky Mountain Spotted Fever Encephalitis

**DOI:** 10.1155/2020/5329420

**Published:** 2020-05-01

**Authors:** Steven D. Rhodes, Alicia M. Teagarden, Brian Graner, Riad Lutfi, Chandy C. John

**Affiliations:** ^1^Department of Pediatrics, Indiana University School of Medicine, Indianapolis, IN, USA; ^2^Section of Pediatric Critical Care Medicine, Indianapolis, IN, USA; ^3^Department of Radiology, Indiana University School of Medicine, Indianapolis, IN, USA; ^4^Ryan White Center for Pediatric Infectious Disease and Global Health, Indiana University School of Medicine, Indianapolis, Indiana, USA

## Abstract

A two-year-old female presented with acutely altered mental status following eight days of fever and rash. She had been camping at an Indiana campground 11 days prior to the onset of illness and was evaluated twice for her fever and rash prior to admission. Laboratory evaluation on admission revealed thrombocytopenia, hyponatremia, and elevated transaminases. The patient developed diffuse cerebral edema, and despite intensive care, the edema led to brain death from Rocky Mountain spotted fever (RMSF). We present this case to highlight the importance of considering RMSF and other tick-borne illnesses in a child with prolonged fever and rash in a nonendemic area and also the difficulty of diagnosis in early stages of disease. A detailed travel history, evaluation of key laboratory findings (white blood count, platelet count, and transaminases), and close follow-up if rash and fevers persist may help to improve detection of RMSF. If a tick-borne illness such as RMSF is suspected, empiric doxycycline therapy should be started immediately, as lab confirmation may take several days and mortality increases greatly after five days of symptoms.

## 1. Introduction

The incidence of spotted fever rickettsiosis, including Rocky Mountain spotted fever (RMSF), a tick-borne illness caused by the organism *Rickettsia rickettsii*, has increased markedly during the last two decades [1, 2]. We present a case of RMSF that sadly ended in the death of the child, to highlight (1) the importance of considering RMSF in a febrile child with a persistent and atypical rash; (2) the difficulty of early diagnosis in areas in which RMSF is uncommon due to the overlap in signs, symptoms, and lab findings between RMSF and common viral illnesses; (3) the clues from history, exam, and lab findings that may increase suspicion of RMSF; and (4) the importance of early treatment in suspected RMSF.

## 2. Case Presentation

A two-year-old previously healthy female developed fevers 11 days after she went on an overnight camping trip to Greencastle, Indiana, in the month of May. One day later, a rash developed on her chest and upper shoulders. She was seen at an emergency department (ED) on day two of her illness and was noted to have a diffuse maculopapular rash on the legs and trunk, with sparing of the palms, soles, and face. Her complete metabolic profile revealed mildly elevated transaminases with an alanine aminotransferase (ALT) of 88 U/L (normal range 7-52 U/L) and aspartate amino transferase (AST) of 127 U/L (normal range 13-39 U/L). Complete blood count values and the remainder of the metabolic profile were within normal limits. The rash was sufficiently unusual that a pediatric hospitalist was called to assess it, but by the time the hospitalist arrived, the rash had resolved. The patient was diagnosed as having a viral syndrome and prescribed ibuprofen for fever control.

She presented to a second ED four days later (day 6 of illness) with persistent fever and rash and decreased activity. Exam revealed fever and a diffuse fine, maculopapular rash in an active, alert child in no apparent distress. A throat swab was positive for group A *Streptococcus* antigen. She was prescribed oral amoxicillin for streptococcal pharyngitis with scarlet fever.

**Figure 1:**
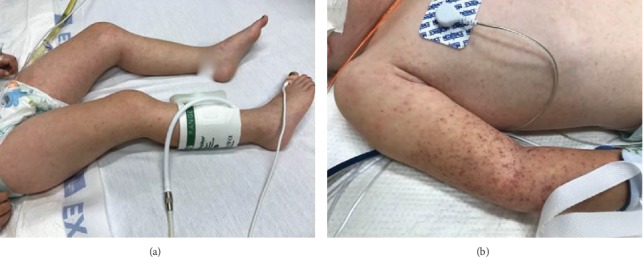
(a) The patient presented with a diffuse, nonblanching maculopapular rash with scattered petechiae and purpura involving the trunk, upper and lower extremities, palms, and soles. (b) Area of petechiae on the arm where a blood pressure cuff was used.

The child continued to have fevers and was progressively less active. Two days later, on the eight day of her illness, she presented to the ED at our medical center with persistent fever, increased rash, lethargy, and inability to ambulate. Physical exam revealed a lethargic child with a temperature of 37.0°C, pulse of 175 beats per minute, respiratory rate of 28 breaths per minute, oxygen saturation of 90% on ambient air, and a blood pressure of 93/55 mmHg. Her exam was notable for impaired consciousness, hypotonia, periorbital edema, and a petechial, nonblanching rash over the all extremities, her trunk, and her palms and soles (Figure 1). She was intubated and admitted to the pediatric intensive care unit. Additional history obtained from the family revealed that she and her father had been camping near a wooded area 11 days prior to the onset of her symptoms, although no tick bites were observed.

Laboratory studies on arrival (day 8 of illness) demonstrated leukocytosis (white blood count, 24 × 10^3^/*μ*L, normal range 4-15 × 10^3^/*μ*L), thrombocytopenia (platelet count, 38 × 10^3^/*μ*L, normal range 150-450 × 10^3^/*μ*L), low fibrinogen (104 mg/dL, normal range 170-399 mg/dL), hyponatremia (132 mmol/L, normal range 135-155 mmol/L), and elevated transaminases (alanine aminotransferase (ALT) 61 U/L; aspartate amino transferase (AST) 141 U/L). A head CT scan without contrast on arrival demonstrated no acute intracranial abnormalities. A sepsis workup was performed, and the patient was started on ceftriaxone, vancomycin, acyclovir, and doxycycline. A lumbar puncture performed after platelet transfusion (day 9 of illness) demonstrated clear fluid, total nucleated cells of 7/*μ*L (normal range 0-7 cells/*μ*L), a glucose of 75 mg/dL (normal range 45-75 mg/dL), and elevated protein at 389 mg/dL (normal range 15-40 mg/dL). A broad workup for infectious pathogens was sent, including for tick-borne diseases.

**Figure 2:**
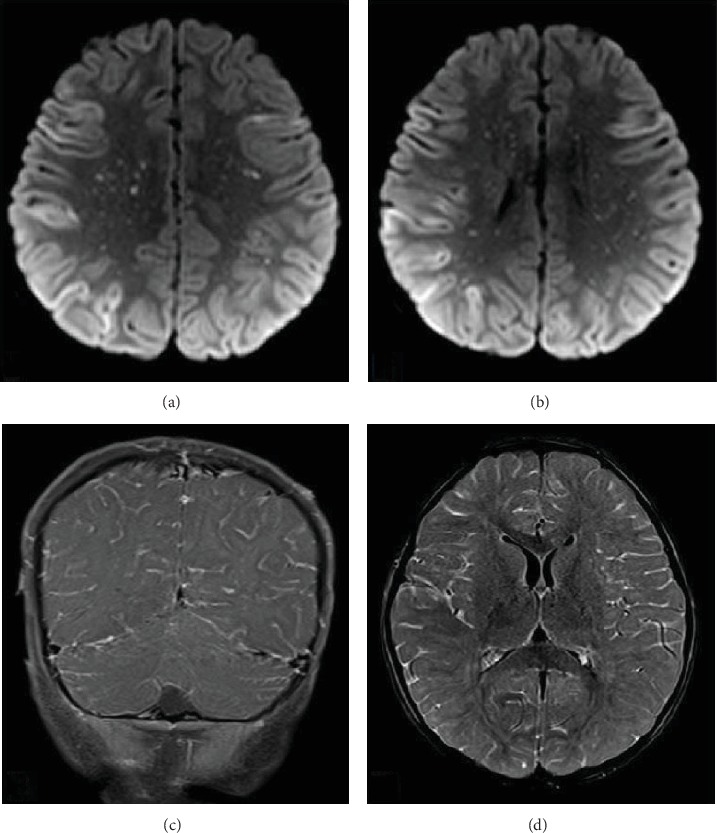
(a, b) Axial diffusion-weighted images demonstrate multifocal areas of gyriform diffusion restriction involving the cortex. Additional scattered foci of diffusion restriction within the periventricular white matter give the appearance of a “starry sky.” (c) A coronal post contrast T1 image demonstrates diffuse leptomeningeal enhancement. (d) An axial post contrast T2 FLAIR image also demonstrates diffuse leptomeningeal enhancement.

Several hours after lumbar puncture, the patient became hyperreflexic, with extensor posturing of all extremities in response to noxious stimulation. A video EEG demonstrated Periodic Lateralized Epileptiform Discharges (PLEDs). The patient was treated with levetiracetam. MRI of the brain revealed diffuse abnormal leptomeningeal enhancement with numerous focal areas of restricted diffusion scattered throughout the gray and white matter of both cerebral hemispheres (Figure 2). A few hours later, her pupils were fixed and dilated, and her EEG demonstrated diffuse attenuation of all activity. Hyperosmolar therapy was administered. An emergent noncontrasted head CT scan demonstrated diffuse cerebral edema and diminished gray-white matter differentiation with impending herniation of the cerebellar tonsils through the foramen magnum. After an examination consistent with brain death, followed by a cerebral brain flow study that also showed no intracranial blood flow, support was withdrawn prior to her second brain death exam based on her family's wishes.

High quantities of *R. rickettsii* DNA on peripheral blood real-time PCR were reported 8 days after the study was obtained on the ninth day of illness. A positive IgM titer (1 : 64, normal range < 1 : 64) and undetectable IgG titer for *R. rickettsii* were reported 6 days after the studies were obtained.

## 3. Discussion

Rocky Mountain spotted fever (RMSF), if treated late in the illness, can lead to potentially fatal complications such as development of severe cerebral edema [3]. For this reason, early treatment is essential. However, early diagnosis can be difficult, particularly in areas where RMSF is not common. Reviewing the case and the literature, we propose potential clues from the history, physical exam, and lab findings that may be useful in identifying higher risk cases that deserve early intervention or close follow-up.

The incidence of spotted fever rickettsiosis (including RMSF) has increased during the last decade, from less than 2 cases per million persons in 2000 to over 11 cases per million persons in 2014 [4]. Illness occurs most frequently in the summer months and six states (Tennessee, Delaware, Missouri, Arkansas, North Carolina, and Oklahoma) account for >60% of cases. Indiana had only 30 cases in 2015 [5], so many providers in the state are not familiar with the illness. The diagnosis of RMSF can be challenging even in areas with more frequent RMSF, as the clinical presentation can resemble that of many other infectious and noninfectious conditions [6, 7].

Since fever and rash are among the most common reasons that parents seek medical attention for their child [8] and overtreatment of these children with doxycycline for suspected RMSF is undesirable, when should one evaluate and treat for RMSF in a child with fever and a rash? While there are no absolute answers, some clinical clues may increase suspicion of RMSF and prompt early empiric treatment. Clues from patient history that may increase suspicion of RMSF include a travel history of camping in wooded areas. However, since RMSF can occur even in urban areas [9], lack of a camping or hiking history does not exclude the diagnosis. Persistence of both fever and rash should also increase suspicion of RMSF, as many viral- and bacterial-associated rashes resolve over a few days, even when fevers persist. The presentation of the rash, including a classic “outward to inward” progression and the presence of the rash on palms and soles, can further support the diagnosis. However, many patients will not have the classic rash progression (e.g., the case patient did not), and some patients have no rash at all. In addition, other infections can cause rashes on the palms and soles, so this sign supports, but is not pathognomonic for RMSF. Common laboratory findings in RMSF include hyponatremia, lymphopenia, thrombocytopenia, coagulopathy, and transaminitis [10]. All of these findings were present in the case patient at the time of hospitalization, but many occur only late in the disease process, as they did in this patient. The caveats mentioned illustrate why early diagnosis of RMSF is often difficult. Prior case series and the findings in the present case report suggest that findings which by themselves may be unremarkable, e.g., a history of camping, elevation of transaminases, and persistence of fevers and rash together for more than 4 days, should in conjunction increase clinical suspicion for RMSF.

The differential for RMSF includes viral infections, such as parvovirus B19, adenovirus, Epstein-Barr virus, and measles; other bacterial illnesses, including sepsis and toxic shock syndrome; other tick-borne illnesses such as *Ehrlichia* and *Anaplasma* infections; and noninfectious illnesses such as Kawasaki disease and systemic juvenile idiopathic arthritis [11].

Routine diagnosis of RMSF is by indirect fluorescent antibody (IFA) testing for IgM and IgG antibodies to *Rickettsia rickettsii*. IgG and IgM antibodies generally rise concurrently during acute illness. However, the first antibody test during acute illness is often negative as insufficient time has elapsed for patients to mount an antibody response. Therefore, testing should be done both acutely and repeated 2 to 6 weeks later. A positive IgM titer is suggestive of acute infection, but IgM titers can remain positive for months, so an IgM titer alone may not establish the diagnosis in a highly endemic area. Recent observations that immunologic reactions can result in frequent false-positive IgM levels (6/13, 46% of patients in one small case series) [12] have led to reconsideration of IgM antibodies in the diagnosis and public health reporting of RMSF. Due to this limitation, IgM no longer contributes to the surveillance case definition as per the 2020 Council State of Territorial Epidemiologists (CTSE) guidelines. A fourfold or greater increase in IgG titer 2-6 weeks after the first testing confirms acute infection with *Rickettsia rickettsii*. Whole blood or serum DNA PCR can confirm the diagnosis early in disease, and PCR testing can also be done on biopsy or autopsy specimens to confirm infection.

Diagnostic imaging is recommended if there is clinical suspicion for central nervous system involvement. CT imaging of the head is insensitive for early changes of meningoencephalitis. CT imaging abnormalities become more apparent as diffuse cerebral edema, infarcts, and associated mass effect progress. As a result, a positive head CT may present too late in the disease process to have a relevant clinical impact. MRI imaging of the brain can better delineate early leptomeningeal inflammation and focal cerebral edema. Contrast-enhanced T1-weighted fat saturated and contrast-enhanced fluid attenuation inversion recovery (FLAIR) sequences further improve sensitivity for leptomeningeal inflammation, although findings of meningoencephalitis and infectious vasculitis can be nonspecific. Diffusion-weighted imaging can provide a useful clue to the diagnosis, with multiple foci of periventricular white matter diffusion restriction in a “starry sky” pattern characteristic for RMSF [13]. This pattern is presumed to arise from perivenular inflammation resulting in focal infarcts and has also been described with CNS viral infections of the genus Henipavirus, a virus carried by bats that is not endemic in the United States [13]. This “starry sky” pattern of white matter diffusion restriction can help distinguish RMSF from other CNS diseases with perivenular inflammation and deposition [13]. Along with the appropriate clinical history, distinctively abnormal MRI findings can help guide early antibiotic intervention.

Empiric treatment with doxycycline, the first-line therapy for RMSF [6, 14], should begin immediately if RMSF is suspected, as the results of definitive laboratory tests including titers and PCR testing may take several days to return and will not be available in time to inform the treatment decision. Provision of doxycycline within 5 days of symptom onset is critical: in one RMSF case series, mortality increased from 6.5% when doxycycline was given within 5 days to 22.9% when given later than 5 days [15], and mortality as low as 0.5% has been reported with treatment in a timely manner [16]. Doxycycline treatment is sometimes delayed due to concerns about the risk of dental staining. However, recent studies have documented no dental staining or enamel hypoplasia following short courses of doxycycline, even after multiple courses were competed [17].

In summary, providers should have a low threshold of suspicion for the diagnosis of RMSF in a child with multiple RMSF risk factors such as a travel history to tick-infested areas, persistence of both fever and rash, specific rash characteristics, and specific lab abnormalities (leukopenia/lymphopenia, thrombocytopenia, hyponatremia, and elevation in transaminases). A child with multiple risk factors, even in nonendemic areas, should receive early empiric doxycycline treatment to prevent severe morbidity and mortality.
